# Medical Legislation and Enforcement of Present Laws against Illegal Practitioners

**Published:** 1908-03

**Authors:** 


### MEDICAL LEGISLATION AND ENFORCEMENT OF PRESENT LAWS AGAINST ILLEGAL PRACTITIONERS.

---

In pursuance of the views expressed in our last issue, in which we urged the importance of physicians protecting their practice, the JOURNAL-RECORD proposes to lend its assistance to the medical profession in driving the charlatans out of the State.

We have placed Dr. Cyrus Strickler, Chairman of the Committee on Legislation of the Fulton County Medical Society, in charge of this department, and urge all of our readers to supply him with any information that may used for such purposes.

We shall also be glad to furnish the State Legislators with marked copies of the JOURNAL-RECORD containing facts regarding any proposed legislation that may be necessary in strengthening our present laws.

Something ought to be done, can be done, and will be done, if the physicians of Georgia will give us a reasonable amount of support in this campaign.

---

Provision has been made in New York whereby the license to practice medicine can be revoked for misdemearors or prefessional mis-deeds, just as lawyers can debar their disreputable members from practicing law. Undoubtedly we should have a similar law in Georgia, and now is the time to start this and other important measures, which should be brought before the State legislature at the next session.

Something certainly can be done to lessen this evil in Atlanta and we urge the Fulton County Medical Society to make a small appropriation to be placed in the hands of the legislative committee, to start the elimination of the charlatans of our own city, who are robbing the citizens of their health and the physicians of their lawful practice.

